# Azacitidine in Chronic Myelomonocytic Leukemia: An Effective and Manageable Approach

**DOI:** 10.4084/MJHID.2014.020

**Published:** 2014-02-16

**Authors:** Andrea Tendas, Luca Cupelli, Agostina Siniscalchi, Laura Scaramucci, Marco Giovannini, Teresa Dentamaro, Alessio Perrotti, Tommaso Caravita, Paolo de Fabritiis, Pasquale Niscola

**Affiliations:** Hematology Division, Sant'Eugenio Hospital, Rome, Italy.

## Abstract

Chronic myelomonocytic leukemia (CMML) is an uncommon neoplastic hematological disorder, typically affecting the elderly, and characterized by a marked clinical heterogeneity and a remarkable propensity for transformation into acute myeloid leukemia. Hypomethylating agents represent the most innovative management approach in this difficult setting. At our institution, between 2010 and 2012, we have treated with azacitidine 10 CMML patients with a median age of 75 (62–86) years. The overall response rate of 70% was achieved without remarkable toxicities; in particular, most therapy-induced side effects were managed on outpatient basis. With a median follow-up of 12,5 (2–27) months, 6 patients are alive, and 4 of them continue to receive the treatment; the median survival from the start of therapy was not reached. In conclusion, also in the light of our encouraging experience, azacitidine can offer new chances of treatment also in the difficult setting of elderly CMML.

## Introduction

Chronic myelomonocytic leukemia (CMML) is an uncommon neoplastic hematological disorder, typically affecting the elderly and characterized by marked clinical heterogeneity and remarkable propensity for transformation into acute myeloid leukemia (AML).^1^ Although CMML portrays a worse outcome when compared to myelodysplastic syndromes (MDS),^2^ it shares several features similar to MDS.^1^,^2^ In particular, affected patients usually are elderly and may present with frailty, comorbidities,^3^ and several forms of disability,^4^ being all factors hampering in most cases the access to aggressive treatment approaches, such as allogeneic hemopoietic stem cells transplantation (HSCT), which still remains the only potentially curative measure in this setting,^1^,^4^ However, the potential disease-modifying activity of hypomethylating agents, such as azacitidine and decitabine, has been recently reported and nowadays these agents represent the most innovative management approach in suitable CMML patients.^1^

## Patients and Methods

At our institution, between 2010 and 2012, we have treated with azacitidine 10 CMML patients (6 males) with a median age of 75 (62–86) years. According to WHO 2008 criteria,^5^ 6, 2 and 2 patients had CMML-2, CMML-1 and AML progressed from CMML respectively. Four patients had proliferative CMML (MPR-CMML) whereas the remaining six presented myelodysplastic CMML (MD-CMML); 2 out of 10 patients had an abnormal karyotype (46, XY, Inv12 and 45, X,-Y, respectively). Details are reported on Table 1. Two patients had secondary likely therapy-related CMML; the first has undergone radio-chemotherapy for a solid tumor 3 years before^6^ whereas the second was an unusual case of aggressive CMML transformed from a 7- years lasting MDS (refractory anemia)^7^. The median time from the diagnosis to the start of azacitidine was 3 (1–21) months. Prior therapies included cytoreductive therapy and erythroid stimulating agents; 4 patients were transfusion dependent at some time point of disease course before starting azacitidine. At the time of treatment, the M.D. Anderson Prognostic Scoring System (MDAPS)^8^ was low, intermediate-1, intermediate-2 and high in 1, 2, 4 and 1 CMML patients, respectively. Criteria for initiation of azacitidine were represented by aggressive disease features, such as splenomegaly, transfusion dependence, cytopenia-related syndromes (bleeding and recurrent infections), an increasing number of peripheral and/or bone marrow blasts, and by patient’s symptomatology, such as general malaise, weight loss. In particular, 2 patients with MPR-CMML-2 were treated because of high bone marrow blasts count (16–19%), 1 patient with MPR-CMML-1 presented severely symptomatic thrombocytopenia and 1 patient with MD-CMML-1 presented severe neutropenia. After written consent had been obtained, all patients received azacitidine (75 mg/m^2^ × 7 days, 5+2+2 schedule, every four weeks, subcutaneously). In MPR-CMML patients undergoing cytoreductive treatment, hydroxyurea was discontinued at the moment of azacitidine start; no cytoreduction was added during treatment and at response assessment. Supportive care was given as required. Bone marrow response was assessed in 9 patients (following the sixth cycle in 6 patients and the fourth in 3); response was not assessed in 1 patient, due to early sudden death (multi-organ failure), which occurred after the second cycle. Responses were classified according to the modified IWG response criteria in myelodysplasia^9^ for MD-CMML and IWG consensus criteria for treatment response in myelofibrosis^10^ for MPR-CMML.

## Results

Out of 9 evaluable patients, 4 (44.4%) patients achieved complete remission (CR) and 3 (33.3%) partial remissions (PR) with an overall response rate (ORR) of 77.7%; 2 (22, 3%) patients maintained a stable disease. At the time of writing (August 2013), the median follow-up was of 12.5 (2 – 27) months; 6 (66,6%) out of 9 evaluable patients were alive, and 4 of them were on azacitidine. Median overall survival (OS) was not reached. Responding patients continued the treatment up to disease progression or intolerance. Two (22.3%) out of 9 CMML patients progressed to AML following the sixth and the fourteenth cycle respectively, after having obtained a CR. Overall, 4 (40%) out of 10 patients initially treated with azacitidine have deceased: 2, 1 and 1 because of AML progression, multi-organ failure (before response assessment) and sudden cardiac death (being the patient with stable CMML). Treatment outcome was similar in MPR-CMML, when compared with MD-CMML; intermediate-2 / high MDAPS CMML and AML patients exhibited a better response rate (CR in 4 and PR in 2 patients, respectively), when compared with low / intermediate-1 MDAPS CMML patients (PR in 1 and stable disease in 2 patients, respectively); treatment outcome details are reported on Table 1. Treatment was well-tolerated, and no remarkable side effects directly attributable to the agent were recorded; in particular, no hospitalization was needed for treatment complications and all of them were managed in the ambulatory setting.

## Conclusion

Our experience was encouraging, mainly due to the following reasons.

Firstly, the use of azacitidine in our hands achieved good responses in 70% of the treated patients, similar to response rate reported in the literature;^11^–^13^ furthermore this result was obtained in high risk patients with unfavorable prognostic profile.

Secondly, this agent was particularly safe and manageable in our experience, and then can be offered also to very old patients.^13^

In conclusion, CMML is a rare disorder and thus there is a paucity of randomized trials utilizing different agents. Until now, most of these patients have received only palliative cytoreduction, being very few of them eligible for more aggressive treatments, such as HSCT. In this view, we have reported a real life experience regarding a little series of CMML patients treated outside a clinical trial. Our experience adds further data to the scanty and scarce evidences supporting the effective activity and the significant benefits provided by azacitidine in this setting. Although our not controlled experience was limited to a small group of patients, our results are encouraging and confirmed previously published papers which reported the possibility of CR with an ORR ranging from 39% to 60% and a median OS from 12 to 37 months.^12^–^13^ Thus, this agent can offer new chances of treatment also in the difficult setting of elderly CMML patients.

## Figures and Tables

**Table 1:** Patients’ series details (diagnosis, treatment response and follow-up data).

Patient	Sex	Age (years)	Disease status at Aza beginning	Karyotype	MDAPS	BM blasts (%)	ECOG	TD	Response	Aza cycles at response	Loss of response (months of response duration)	FU (months)	Status	Cause of death	Disease status at FU	Total Aza cycles at FU
1	M	83	AML	Normal	NA	30	2	Yes	NE	NA	NA	2	Death	MOF	NE	2
2	M	76	CMML-2 (MPR)*	Normal	Int-2	19	1	No	PR°°	6	No	19	Alive	NA	PR	13
3	M	72	CMML-1 (MPR) **	Normal	Low	3	1	No	SD°°	6	No	12	Death	Sudden cardiac death	SD	12
4	F	72	CMML-2 (MD) ***	Normal	Int-1	11	1	No	SD	4	No	27	Alive	NA	SD	20
5	M	80	CMML-1 (MD)	Normal	Int-1	8	0	Yes	PR	6	No	18	Alive	NA	PR	14
6	M	86	CMML-2 (MPR) *	Normal	Int-2	18	1	No	PR°°	6	No	16	Alive	NA	PR	12
7	M	75	AML	46, XY, Inv12	NA	30	1	Yes	CR	6	Yes (5)	13	Death	AML	AML	14
8	M	62	CMML-2 (MD)	Normal	High	16	1	No	CR	4	Yes (1)	8	Death	AML	AML	6
9	F	62	CMML-2 (MPR) *	Normal	Int-2	16	0	No	CR°°	6	No	10	Alive	NA	CR	9
10	M	82	CMML-2 (MD)	45, X, -Y	Int-2	12	1	Yes	CR	4	No	10	Alive	NA	CR	10

Aza: azacitidine; AML: acute myeloid leukemia; CMML: chronic myelomonocytic leukemia; MPR: myeloproliferative; MD: myelodysplastic; ECOG: Eastern Cooperative Oncology score, TD: transfusion dependence; NA: not applicable; NE: not evaluated; PR: partial response; SD: stable disease; CR: complete response; FU: follow-up; MOF: multi-organ failure.

* Indication for treatment was high bone marrow blasts count (16–19%);

** Indication for treatment was severe symptomatic thrombocytopenia;

*** Indication for treatment was severe symptomatic neutropenia;

°Response criteria: Responses were classified according to the modified IWG response criteria in myelodysplasia^9^ for MD-CMML and IWG consensus criteria for treatment response in myelofibrosis^10^ for MPR-CMML;

°°In MPR-CMML patients, ongoing cytoreductive treatment was discontinued at the moment of azacitidine start; no cytoreduction was added during treatment and at response assessment.
